# Detection of high cardiovascular risk patients with ankylosing spondylitis based on the assessment of abdominal aortic calcium as compared to carotid ultrasound

**DOI:** 10.1186/s13075-018-1684-y

**Published:** 2018-08-29

**Authors:** Javier Rueda-Gotor, Fernanda Genre, Alfonso Corrales, Ricardo Blanco, Patricia Fuentevilla, Virginia Portilla, Rosa Expósito, Cristina Mata, Trinitario Pina, Carlos González-Juanatey, Luis Rodriguez-Rodriguez, Miguel A. González-Gay

**Affiliations:** 1 Epidemiology, Genetics and Atherosclerosis Research Group on Systemic Inflammatory Diseases, Division of Rheumatology, Hospital Universitario Marqués de Valdecilla, IDIVAL, University of Cantabria, Avenida de Valdecilla, s/n, 39008 Santander, Spain; 2 Division of Rheumatology, Hospital Comarcal, Laredo, Cantabria Spain; 3 Division of Cardiology, Hospital Lucus Augusti, Lugo, Spain; 4 Division of Rheumatology, Instituto de Investigación Sanitaria del Hospital Clínico San Carlos (IDISSC), Hospital Clínico San Carlos, Madrid, Spain; 5 Cardiovascular Pathophysiology and Genomics Research Unit, School of Physiology, Faculty of Health Sciences, University of the Witwatersrand, Johannesburg, South Africa

**Keywords:** Ankylosing spondylitis, Cardiovascular disease, Abdominal aortic calcification, Lumbar lateral spine radiography, Carotid ultrasonography

## Abstract

**Background:**

This study aimed to determine whether, besides carotid ultrasound (US), a lateral lumbar spine radiography may also help identify ankylosing spondylitis (AS) patients at high risk of cardiovascular (CV) disease.

**Methods:**

A set of 125 AS patients older than 35 years without a history of CV events, diabetes mellitus, or chronic kidney disease was recruited. Carotid US and lateral lumbar spine radiography were performed in all of them. The CV risk was calculated according to the total cholesterol systematic coronary risk evaluation (TC-SCORE) algorithm. Presence of carotid plaques was defined following the Mannheim Carotid Intima-media Thickness and Plaque Consensus. Abdominal aortic calcium (AAC) in a plain radiography was defined as calcific densities visible in an area parallel and anterior to the lumbar spine.

**Results:**

Carotid US showed higher sensitivity than lateral lumbar spine radiography to detect high CV risk in the 54 patients with moderate TC-SCORE (61% versus 38.9%). Using carotid plaques as the gold standard test, a predictive model that included a TC-SCORE ≥ 5% or the presence of AAC in the lateral lumbar spine radiography in patients with both moderate and low CV risk (< 5%) according to the TC-SCORE yielded a sensitivity of 50.9% with a specificity of 95.7% to identify high/very high CV-risk AS patients. A positive correlation between AAC and carotid plaques was observed (*r*^2^ = 0.49, *p* < 0.001).

**Conclusions:**

A lateral lumbar spine radiography is a useful tool to identify patients with AS at high risk of CV disease.

## Background

As occurs with other chronic inflammatory conditions, ankylosing spondylitis (AS) is associated with a process of accelerated atherosclerosis [1, 2] which leads to increased rates of subclinical atherosclerosis [3] and cardiovascular (CV) events [4]. A recent meta-analysis of seven longitudinal studies reported an increased frequency of myocardial infarction (odds ratio (OR) 1.60, 95% confidence interval (CI) 1.32–1.93) and stroke (OR 1.50, 95% CI 1.39–1.62) in AS patients when compared to the general population [4]. In a population-based study which included 21,473 AS patients and 86,606 controls matched for age, sex, and location of residence, Haroon et al. [5] found a 36% higher risk of vascular mortality in AS.

Primary prevention strategies designed to avoid atherosclerosis-related CV events in the general population are based on the identification of individuals at high CV risk who can benefit from appropriate prevention measures [6]. In this regard, statin use has proved to be very effective, being able to reduce overall mortality by 15% [7]. The benefit may be even greater in AS patients according to a recent study which found a 37% lower risk of all-cause mortality associated with statin initiation [8].

The 2016 “European Guidelines on CV disease prevention in clinical practice” recommended using the total cholesterol systematic coronary risk evaluation (TC-SCORE) to stratify the CV risk and to identify individuals at high CV risk who were candidates for treatment [6]. The TC-SCORE predicts the individual’s absolute risk for fatal CV events considering age, sex, total cholesterol levels, smoking, and blood pressure, stratifying the CV risk into low (< 1%), moderate (≥ 1 and < 5%), high (≥ 5 and < 10%), and very high (≥ 10%).

Unfortunately, predictive models designed for the general population do not consider the inflammatory process as a proatherogenic factor, leading to an underestimation of the CV risk when used in inflammatory diseases like AS. In this regard, a recent study disclosed in AS patients a 10-year cumulative incidence of CV events three times higher than that predicted based on the Framingham Risk Score (FRS) [9].

In an attempt to improve the TC-SCORE predictions, the 2016 European Society of Cardiology (ESC) guidelines proposed the use of different imaging techniques to identify high-CV risk individuals with subclinical atherosclerosis [6]. Carotid ultrasonography (US) and multidetector coronary tomography (MDCT) allow one to detect, respectively, carotid plaques and coronary calcifications, which are considered independent predictors of CV events capable of providing additional value to the FRS when estimating CV risk [10, 11]. According to the 2016 ESC guidelines, the presence of both surrogate markers of atherosclerosis automatically implies a very high CV risk [6]. Our group has recently demonstrated that carotid US and MDCT are very useful to redefine the CV risk in AS: up to 63% and 30% of our AS patients with moderate CV risk according to the SCORE risk charts had carotid plaques and coronary calcifications, respectively [12]. However, the restricted availability of MDCT in the daily clinical practice and the considerable amount of radiation associated with the use of this technique may be limitations for to its generalized use.

Abdominal aortic calcium (AAC) constitutes another surrogate marker of atherosclerosis easily detectable in a lateral lumbar spine radiography [13]. Unlike MDTC, it is available in most AS patients. Aortic calcification has been demonstrated to represent true intimal atherosclerosis in postmortem studies [14], correlating with the degree of atherosclerosis in the coronary arterial beds [15]. As occurs with carotid plaques and coronary calcification, AAC deposits constitute an independent predictor of subsequent vascular morbidity and mortality capable of improving the risk prediction based on the FRS [16].

Taking all these considerations into account, the purpose of the present study is to determine whether, besides carotid US, the presence of ACC deposits detected by plain lumbar spine radiographs is able to improve the identification of those AS patients with high/very high CV risk who, therefore, would be candidates to receive intensive preventive therapy.

## Methods

### Patients

In this cross-sectional study, a set of 125 consecutive AS patients seen over a 5-year period at Hospital Universitario Marqués de Valdecilla and Hospital de Laredo (Cantabria, northern Spain) who fulfilled definitions for AS according to the 1984 modified New York criteria [17] were recruited. Patients with a history of CV events (ischemic heart disease, cerebrovascular accident, peripheral arterial disease, or heart failure) were excluded. This was also the case for those with type 2 diabetes mellitus or with two fasting plasma glucose levels on different days at the time of disease diagnosis or over the extended follow-up > 125 mg/dl as well as those with chronic kidney disease (glomerular filtration rate < 60 ml/min/1.73 m^2^) because they are considered as having high or very high CV risk according to current guidelines.

Two clinical indexes of disease activity (Bath Ankylosing Spondylitis Disease Activity Index (BASDAI) and Ankylosing Spondylitis Disease Activity Score (ASDAS)), a functional status index (Bath Ankylosing Spondylitis Functional Index (BASFI)), a metrologic index (Bath Ankylosing Spondylitis Metrology Index (BASMI)), and an enthesitis index (Maastricht Ankylosing Spondylitis Enthesitis Score (MASES)) were evaluated in all patients at the time of the carotid US assessment [18–22].

Information on history of hip involvement, synovitis, enthesitis, extra-articular manifestations (anterior uveitis, psoriasis, and inflammatory bowel disease), syndesmophytes, and HLA-B27 status was also assessed. This was also the case for data on family history of early CV events in first-degree relatives, waist circumference, body mass index, blood pressure at the time of study, and history of traditional CV risk factors (smoking, hypertension, dyslipidemia, and obesity).

Data on C-reactive protein (CRP) and the erythrocyte sedimentation rate (ESR) at the time of recruitment and at disease diagnosis, identification of patients with CRP serum levels higher than 3 mg/L at the time of diagnosis, and total cholesterol, HDL-cholesterol, LDL-cholesterol, and triglycerides at the time of the study were also assessed. Information on therapy including treatment with anti-tumor necrosis factor (anti-TNF) alpha agents from the disease diagnosis was also reviewed.

The TC-SCORE system estimates the 10-year risk of a first fatal atherosclerotic event, whether heart attack, stroke, or other occlusive arterial disease, including sudden cardiac death. Risk estimates have been produced as charts for high and low-risk regions in Europe [6]. Spain was included in the low-risk region of Europe. The risk factors incorporated in the TC-SCORE are those previously described: age, gender, smoking, total cholesterol levels, and systolic blood pressure. Subjects with TC-SCORE < 1% are included in the category of low risk. Those with a TC-SCORE ≥ 1% and < 5% are in the category of moderate risk. When the chart TC-SCORE result is ≥ 5% and < 10% they are classified as having high risk. Finally, those patients with TC-SCORE results ≥ 10% are included in the category of very high CV risk.

The earliest age at which the CV risk scores should be used in the general population has not been rigorously established. Both European [6] and North American [23] guidelines recommend their application in individuals over 40 years old. However, this cutoff point is not uniform and varies across the different CV risk scores: 30 years old for the FRS [24], 40 years old for the TC-SCORE [6], and 45 years old for the Reynolds Risk Score [25], which can only be applied from this age onward. In Spain, the Framingham-based REGICOR adapted function, a CV risk function validated in the Spanish population [26], established its use at an intermediate point of 35 years old. Since patients with AS have early accelerated atherosclerosis, and we observed carotid plaques in patients under 40 years old, we included in the analysis all patients who were age 35 years and older.

### Carotid US examination

A set of 125 AS patients underwent carotid US examination to detect focal plaques in the extracranial carotid tree. According to the Mannheim Carotid Intima-media Thickness and Plaque Consensus, a carotid plaque was defined as a focal protrusion in the lumen at least cIMT > 1.5 mm, protrusion at least 50% greater than the surrounding cIMT, or arterial lumen encroaching > 0.5 mm [27]. Carotid US was performed using a commercially available scanner (Mylab 70; Esaote, Genoa, Italy) equipped with a 7–12 MHz linear transducer and the automated software-guided radiofrequency technique Quality Intima Media Thickness in real time (QIMT; Esaote, Maastricht, Holland). Patients with carotid plaques were considered as having very high CV risk.

### Evaluation of abdominal aortic calcium deposits

**Fig. 1:**
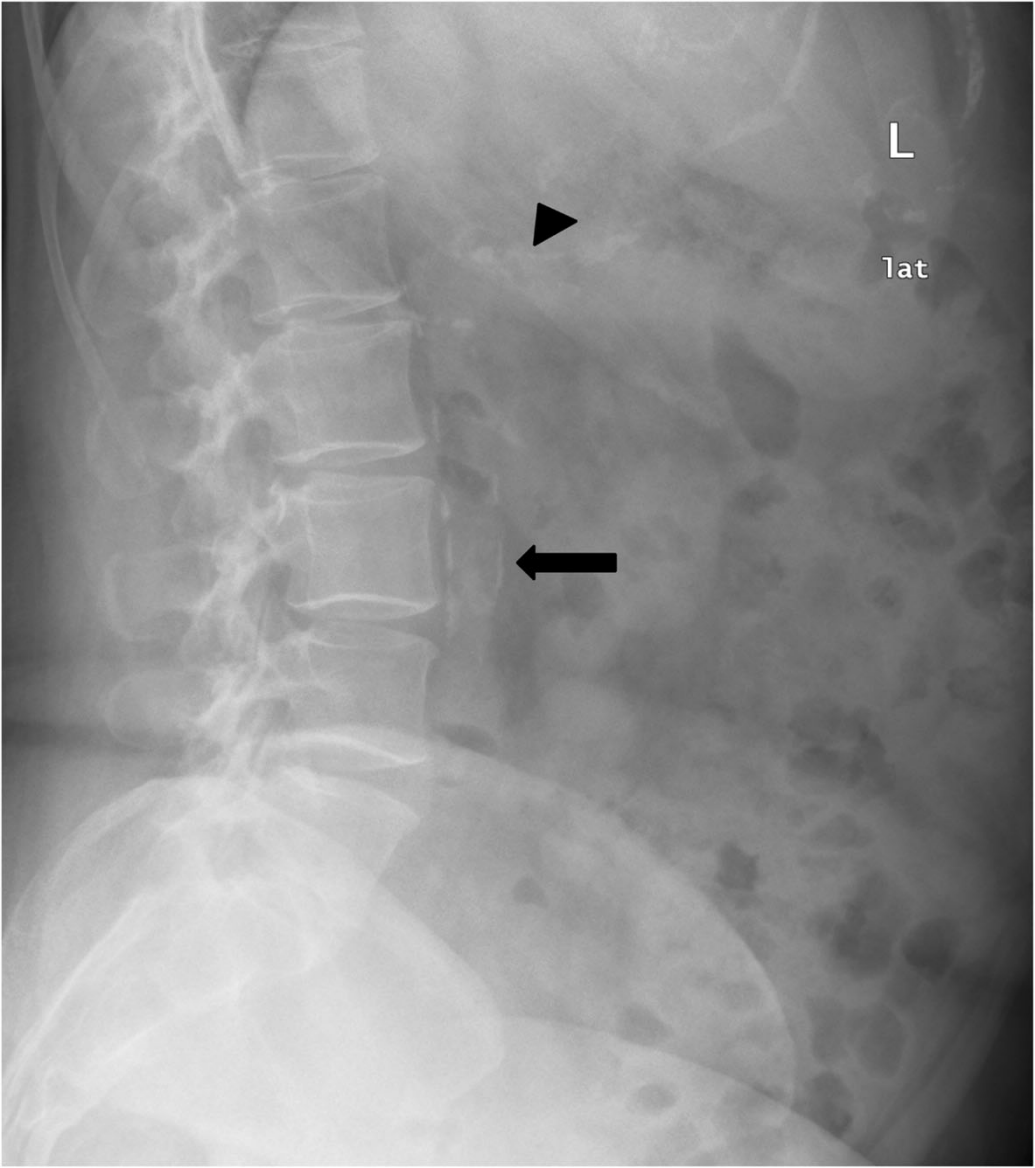
Abdominal aortic calcifications and calcified costal cartilages. Abdominal aortic calcifications seen as calcium deposits localized in area parallel to lumbar spine and anterior to lower part of spine (long arrow). Calcified costal cartilages have a typical pattern distinguishable from aortic calcifications (arrowhead)

The lateral lumbar spine radiography was performed in the standing position and all X-ray scans were evaluated by an independent reader blinded to the participant’s clinical status. We assessed the presence of calcific deposits at each vertebral segment from the first to fourth lumbar vertebrae, both in the posterior and anterior walls of the aorta (Fig. 1). Calcific deposits were regarded as present if densities were visible in an area parallel to the lumbar spine and anterior to the lower part of the spine. Aortic densities at the upper part of the lumbar spine (L1–L2 region) often overlapped the vertebrae as the abdominal aorta in the L1–L2 region is often lateral to the spine. Densities overlapping the vertebrae were deemed present only if they extended from or formed a clear pattern with those of the lower part of the aorta. Since calcific deposits tend to occur first in the lower aorta [28, 29], calcific deposits at the upper levels were almost always accompanied by extensive calcifications in the lower part of the aorta. Other calcific deposits visible in lumbar films, such as intestinal calcifications and calcified costal cartilages, were clearly distinguishable from aortic calcifications (Fig. 1).

The reproducibility of the evaluation of AAC deposits was also evaluated in 20 patients by two investigators (JR-G and AC). The correlation coefficient for AAC was 0.96.

The subject’s written consent was obtained for all of the cases. The study was approved by the local ethical committee.

### Statistical analysis

Categorical variables were described as percentages and quantitative variables as mean ± standard deviation (SD) or median (interquartile range (IQR)).

Correlation between the presence of carotid plaques and lumbar aortic calcification was analyzed using the Pearson correlation. For each CV risk model, sensitivity, specificity, percentage of correctly classified patients, and area under the receiver operating characteristic (ROC) curve (with 95% CI) were estimated.

## Results

### Characteristics of AS patients

The main features of the 125 AS patients included in the study are summarized in Table 1.

**Table 1:** Features of 125 ankylosing spondylitis (AS) patients

Variable	AS (*n* = 125)
Men/women, *n*	78/47
Age at time of study (years), mean ± SD	48.3 ± 9.6
Age at time of diagnosis (years), mean ± SD	39.5 ± 9.6
HLA-B27 positive, *n* (%)	92 (74.8)
Syndesmophytes, *n* (%)	51 (41.5)
History of synovitis, *n* (%)	35 (28.0)
History of enthesitis, *n* (%)	45 (36.0)
Extra-articular manifestations, *n* (%)	38 (30.4)
Psoriasis	12 (9.6)
Inflammatory bowel disease	10 (8.0)
Uveitis	22 (17.6)
Therapy with TNF inhibitors, *n* (%)	49 (39.8)
ASDAS, mean ± SD	2.4 ± 0.9
BASFI, mean ± SD	3.9 ± 2.5
BASMI, mean ± SD	3.2 ± 1.7
MASES, median (IQR)	1 (0.0–4.0)
BASDAI, mean ± SD	3.7 ± 2.0
CRP (mg/l), median (IQR)	
At time of study	5.2 ± 6.7
At time of disease diagnosis	10.8 ± 19.0
CRP > 3 mg/L at time of disease diagnosis, *n* (%)	72 (57.6)
ESR (mm/1st hour), median (IQR)	
At time of study	12.4 ± 13.4
At time of disease diagnosis	16.1 ± 15.9
History of classic cardiovascular risk factors, *n* (%)	
Current smokers	37 (29.6)
Ex-smokers	31 (24.8)
Obesity	23 (18.4)
Dyslipidemia	40 (32.0)
Hypertension	21 (16.8)
Blood pressure (mmHg), mean ± SD	
Systolic	130.6 ± 15.3
Diastolic	79.5 ± 9.7
Cholesterol and triglycerides (mg/dl), mean ± SD	
Total cholesterol	200.7 ± 34.5
HDL cholesterol	55.6 ± 14.8
LDL cholesterol	124.6 ± 31.7
Triglycerides	97.3 ± 49.4
Carotid plaques, *n* (%)	55 (44.0)
Aortic calcification, *n* (%)	28 (22.4)
TC-SCORE ≥ 5, *n* (%)	7 (5.6)

Main epidemiologic, clinical, radiographic, and ultrasonography features of a series of 125 AS patients older than 35 years of age without history of cardiovascular events, diabetes mellitus, or chronic kidney disease

*ASDAS* Ankylosing Spondylitis Disease Activity Score, *BASDAI* Bath Ankylosing Spondylitis Disease Activity Index, *BASFI* Bath Ankylosing Spondylitis Functional Index, *BASMI* Bath Ankylosing Spondylitis Metrology Index, *CRP* C-reactive protein, *ESR* erythrocyte sedimentation rate, *HDL* high-density lipoprotein, *IQR* interquartile range, *LDL* low-density lipoprotein, *MASES* Maastricht Ankylosing Spondylitis Enthesitis Score, *SD* standard deviation *TC-SCORE* total cholesterol systematic coronary risk evaluation, *TNF* tumor necrosis factor

Men outnumbered women (*n* = 78; 62.4%) and the mean ± SD age at the time of the study was 48.3 ± 9.6 years. HLA-B27 was positive in 92 (74.8%) patients, and syndesmophytes were present in 51 (41.5%) patients. Thirty-eight (30.4%) patients had extra-articular manifestations: uveitis, psoriasis, and inflammatory bowel disease were present in 17.6%, 9.6%, and 8% of patients, respectively. The mean ± SD values of the BASDAI and ASDAS were 3.7 ± 2.0 and 2.4 ± 0.9. Seventy-two (57.6%) patients were found to have CRP > 3 mg/L at the time of disease diagnosis. TNF inhibitors were used in 39.8% of cases. Regarding findings of subclinical atherosclerosis, carotid plaques and AAC deposits were found in 55 (44.0%) and 28 (22.4%) patients, respectively. Other characteristics of this series of patients with AS are presented in Table 1.

### TC-SCORE risk groups and severe atherosclerotic disease using carotid US and lateral spine radiography

The CV risk was calculated using the TC-SCORE. Based on this algorithm, patients were classified into four different CV risk categories: low, moderate, high, and very high. Then, the frequency of carotid plaques in each category was calculated to assess the ability of the TC-SCORE to correctly classify patients as having high/very high CV risk (Table 2). We also analyzed the prevalence of AAC in the lateral spine radiography in each group of risk (Table 2). Following this approach, 33 of 54 (61.1%) patients classified as having moderate CV according to the TC-SCORE had plaques when the carotid US was performed, whereas 21 of 54 (38.9%) patients with moderate CV risk according to the TC-SCORE also had AAC in the lateral lumbar spine radiography. Moreover, 23.4% and 4.7% of AS patients who fulfilled the category of low CV risk according to the TC-SCORE had carotid plaques and AAC, respectively (Table 2).

**Table 2:** Prevalence of carotid plaques and abdominal aortic calcium in the different groups of cardiovascular risk

TC-SCORE		Carotid ultrasonography	Lateral lumbar X-ray
		Carotid plaques (*n* = 55, 44%)	AAC deposits (*n* = 28, 22.4%)
Low (< 1%)	*n* = 64	15/64 (23.4%)	3/64 (4.7%)
Moderate (≥ 1% and < 5%)	*n* = 54	33/54 (61.1%)	21/54 (38.9%)
High (≥ 5% and < 10%)	*n* = 7	7/7 (100%)	4/7 (57.1%)
Very high (≥ 10%)	*n* = 0	0 (0%)	0 (0%)

Presence of carotid plaques and AAC deposits in 125 ankylosing spondylitis patients older than 35 years of age without cardiovascular events, diabetes mellitus, or chronic kidney disease, classified according to their cardiovascular risk

*AAC* abdominal aortic calcium, *TC-SCORE* total cholesterol systematic coronary risk evaluation

### Correlation between AAC and carotid US in AS patients

The validity of lateral lumbar spine radiography to identify AS patients at high CV risk was assessed by analyzing the correlation between AAC and carotid plaques (Table 3). We observed that 25 of 28 (89.3%) patients with AAC deposits also had plaques in the carotid US assessment. Regarding patients without AAC deposits in the lateral lumbar spine radiography, 69.1% of them did not have carotid plaques either (Table 3). We observed a positive correlation between AAC and carotid plaques (*r*^2^ = 0.49, *p* < 0.001).

**Table 3:** Correlation between abdominal aortic calcium (AAC) deposits and carotid plaques

Lateral lumbar X-ray		Carotid ultrasonography	
		Presence of carotid plaques (*n* = 55/125, 44%)	Absence of carotid plaques (*n* = 70/125, 56%)
Presence of AAC	*n* = 28 (22.4%)	25/28 (89.3%)	3/28 (10.7)
Absence of AAC	*n* = 97 (77.6%)	30/97 (30.9%)	67/97 (69.1%)

Correlation between the presence of AAC deposits and the presence of carotid plaques in 125 patients with ankylosing spondylitis older than 35 years of age without cardiovascular events, chronic kidney disease, or diabetes mellitus

### Model to establish the presence of high/very high CV risk in patients with AS

Since many patients categorized as having low or moderate CV risk when the TC-SCORE was applied had subclinical atherosclerosis, we set up a predictive model to identify AS patients with high/very high CV risk (Table 4). In this regard, according to the ESC 2016 guidelines, we classified patients as having high/very high CV risk if they had carotid plaques in the carotid US [6]. Following this approach, 55 of 125 patients fulfilled definitions of high/very high CV risk. However, only 7 (12.7%) of them were detected using the TC-SCORE, without the inclusion in the model of results of imaging techniques (model 1). The detection of aortic atherosclerosis by lateral lumbar spine radiography yielded a high specificity (95.7%) with low sensitivity (45.5%) to identify AS patients with very high CV risk (model 2). A predictive model that included a TC-SCORE ≥ 5% or the presence of AAC in lateral lumbar spine radiography in patients with moderate CV risk according to the SCORE (≥ 1% and < 5%) (model 3) showed the same results as those found in model 2. A higher sensitivity (50.9%) with the same specificity (95.7%) was achieved when the predictive model included TC-SCORE ≥ 5% or the presence of AAC in the lateral lumbar spine radiography in patients with both moderate and low CV risk according to the TC-SCORE (< 5%) (model 4).

**Table 4:** Diagnostic models designed to identify ankylosing spondylitis patients with very high cardiovascular risk using presence of carotid plaques as the gold standard test

	Sensitivity (%)	Specificity (%)	Correctly classified (%)	ROC (95% CI)
Model 1. TC-SCORE ≥ 5%	12.7	100	61.6	0.56 (0.52–0.61)
Model 2. Lateral lumbar spine radiography (presence of AAC)	45.5	95.7	73.6	0.71 (0.64–0.78)
Model 3. TC-SCORE ≥ 5% or TC-SCORE ≥ 1% and < 5% plus lateral lumbar spine radiography (presence of AAC)	45.5	95.7	73.6	0.71 (0.64–0.78)
Model 4. TC-SCORE ≥ 5% or TC-SCORE < 5% plus lateral lumbar spine radiography (presence of AAC)	50.9	95.7	76.0	0.73 (0.66–0.80)

*AAC* abdominal aortic calcium, *CI* confidence interval, *ROC* receiver operating characteristic, *TC-SCORE* total cholesterol systematic coronary risk evaluation

## Discussion

To the best of our knowledge this is the first study that aimed to assess the ability of the lateral lumbar spine radiography, a diagnostic tool widely available in AS patients, to improve the CV risk stratification in patients with AS. We observed that the presence of AAC deposits detected by a lateral lumbar spine radiography allows one to identify individuals with AS at high risk of CV disease. In this regard, in the present series of 125 AS patients without CV events, chronic kidney disease, or diabetes mellitus, 21 of 54 (38.9%) patients with moderate TC-SCORE and 3 of 64 (4.7%) patients with low TC-SCORE showed AAC in the lateral lumbar spine radiography.

The presence of AAC in a plain radiography has been reported to be associated with an increased risk of CV events, CV mortality [13], and congestive heart failure [30] in the Framingham cohort. Overall, AAC adds to the prediction for intermittent claudication (IC), ischemic stroke (IS), and coronary heart disease over and above traditional CV risk factors in patients with low and intermediate risk [16]. AAC deposits also demonstrated to be a predictor of incident stroke in the Rotterdam study, even stronger than carotid plaques [31]. Both the Rotterdam and the Framingham studies used a quantitative scale to grade the severity of the aortic calcium deposits. It is worth noting that, even though the increase of CV risk was found to be proportional to the extent of the calcifications, AAC demonstrated to be a predictor of CV events and mortality also in cases of reduced calcifications. In keeping with these findings, in a Netherlands population Witteman et al. [32] found a strong independent association between CV deaths and aortic calcifications of any size, regardless of the extent of the calcific deposits. A postmortem study that analyzed the accuracy of the radiologic AAC for the diagnosis of true aortic atherosclerosis confirmed that even the smaller densities indicated the presence of advanced atheromatous plaques, which were almost invariably ulcerated [14]. Witteman et al. [32] also disclosed a high predictive role of AAC for CV death in young individuals, with a six-fold increased risk in men aged 45 years independently of the major CV risk factors, and no excess risk at age 75 years. These findings are particularly relevant in AS patients since the disease typically starts before the age of 45 years and the TC-SCORE tends to underestimate the CV risk, especially in this age group in which practically all patients are classified as having low or moderate CV risk. Indeed, the mean age in our series was 48 years and 118 of the 125 (94%) AS patients were found to have a low or moderate TC-SCORE.

The 2014 ESC guidelines on the diagnosis and treatment of aortic diseases [33] recommended adopting general preventive measures to control risk factors in the presence of aortic atherosclerosis. Treatment with statins led to regression of thoracic aortic and retardation of abdominal aortic atheroma burden assessed by magnetic resonance imaging [34], as well as reduction of the inflammation assessed by PET [35]. In a retrospective study of 519 patients with severe aortic plaque seen on transesophageal echocardiography, only statin treatment was associated with a 70% lower risk of events [36]. A correlation between AAC and atherosclerotic findings in the carotid and coronary arterial beds has previously been reported [15, 37]. This finding constitutes an additional argument to support statin use in AS patients with AAC. The presence of carotid plaques, considered a surrogate marker of subclinical atherosclerosis in the 2016 ESC guidelines, automatically implies a very high CV risk with the consequent indication of statin use. Taking into account these considerations, we can conclude that almost 50% of AS included in the categories of low and moderate CV risk according to the TC-SCORE risk algorithm would benefit from additional primary prevention measures, with special emphasis on the use of statins, if a plain lateral lumbar spine radiography is performed. This is a crucial aspect considering that statins have been shown to decrease mortality by 37% in AS, a figure double that observed in the general population [8].

The major limitation of our study was the absence of prospective follow-up data for the studied patients. Because of this, we dealt with a surrogate outcome based on extrapolation of the data that might not be applicable to AS.

In an attempt to elucidate the best strategy to identify AS patients at high CV risk, we assessed the ability of lateral lumbar spine radiography to detect severe findings of subclinical atherosclerosis. A predictive model that included a TC-SCORE ≥ 5% or the presence of AAC in lateral lumbar spine radiography in patients with a low or moderate TC-SCORE (TC-SCORE < 5%) allowed us to detect half of the AS patients with high/very high CV-risk, also demonstrating a high degree of specificity (95.7%).

## Conclusions

When carotid US is not available, plain lateral lumbar spine radiography can be an easy and affordable tool to identify AS patients at high CV risk.

## Abbreviations

AAC: Abdominal aortic calcium
AS: Ankylosing spondylitis
ASDAS: Ankylosing Spondylitis Disease Activity Score
BASDAI: Bath Ankylosing Spondylitis Disease Activity Index
BASFI: Bath Ankylosing Spondylitis Functional Index
BASMI: Bath Ankylosing Spondylitis Metrology Index
CRP: C-reactive protein
CV: Cardiovascular
ESR: Erythrocyte sedimentation rate
FRS: Framingham Risk Score
IC: Intermittent claudication
IQR: Interquartile range
IS: Ischemic stroke
MASES: Maastricht Ankylosing Spondylitis Enthesitis Score
MDCT: Multidetector coronary tomography
OR: Odds ratio
ROC: Receiver operating characteristic
SD: Standard deviation
TC-SCORE: Total cholesterol systematic coronary risk evaluation
TNF: Tumor necrosis factor
US: Ultrasonography
